# Quality Improvement of GaN Epi-layers Grown with a Strain-Releasing Scheme on Suspended Ultrathin Si Nanofilm Substrate

**DOI:** 10.1186/s11671-022-03732-1

**Published:** 2022-10-15

**Authors:** Kejia Wang, Yuzi Song, Yichun Zhang, Yunyan Zhang, Zhiyuan Cheng

**Affiliations:** School of Micro-Nanoelectronics, Zhejiang University, Hangzhou, 311200 China

**Keywords:** Nitrides, Compliant substrates, Epitaxy, TEM

## Abstract

The material quality of III-nitrides is severely limited by the lack of cost-effective substrates with suitable lattice and thermal expansion coefficients. A suspended ultrathin silicon membrane substrate ($\sim$16 nm), fabricated by an easy process on SOI substrates, is thus designed for nitride epitaxial growth, which can effectively release the strain in the epi-layers, and has demonstrated large-area (Al)GaN growth with a smooth surface and greatly reduced defect density. This research provides a promising CMOS-compatible method for growing cost-effectively high-quality III-nitrides that can be used for the development of high-performance devices.

## Introduction

III-nitride semiconductor materials with a wide bandgap are promising for various applications, such as high frequency and power electronic devices, and optoelectronics like lasers and light-emitting diodes [1–3]. However, owing to the high-cost of native substrates, commercial nitride materials are generally grown through hetero-epitaxy on foreign substrates, including sapphire, SiC and Si [4]. Si substrates have received particular attention because of its low material cost, wide use in semiconductor industry, and potential for integration of nitride devices with Si devices and Si electronics. However, hetero-epitaxial growth of III-nitride materials on Si commonly exhibit remnant strain and high threading dislocation density (TDD) due to various issues, especially large lattice mismatch and thermal expansion coefficient mismatches [5–9].

**Fig. 1:**
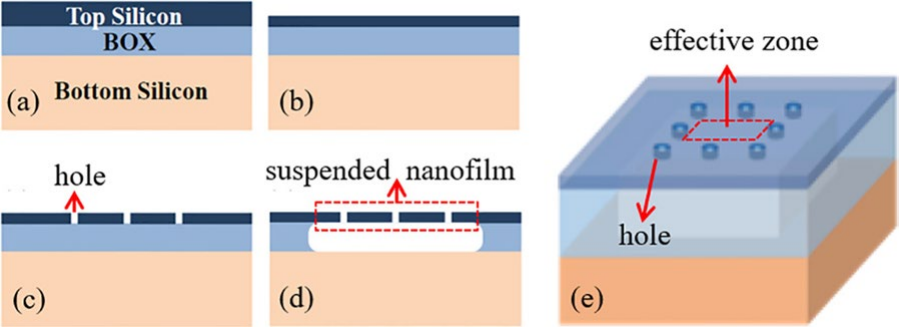
Fabrication process schematic of the suspended ultrathin samples: **a** commercial SOI structure, **b** thinning of top Si film, **c** etching of holes on the top Si film, **d** removal of underneath BOX layer by undercut etching process, **e** 3D schematic of **d**, and the effectively suspended zone is enclosed by the red dashed-lines

The reduction in TDD in these III-nitride films on Si is one of the most critical objectives for achieving high-performance devices, and thus great effort has been made to improve the quality of nitride epi-layers [10–14]. Among the methods, compliant substrate is highly promising as it can reduce the stress caused by mismatches and at the same time allow for the incorporation of nitride devices into silicon-based integrated circuits [15–20]. SOI is one of the most studied compliant substrates that is readily available commercially. However, the buried oxide layer (BOX) restrains the flexibility of top silicon layer (Fig. 1a) and may introduce extra thermal stress during cooling process [21–23]. Besides, according to the critical thickness condition theory, if the substrate thickness is less than its critical thickness of relaxation, the substrate can allow the growth of infinitely thick epilayers without misfit dislocations [24–26]. To be an efficient compliant substrate, the top silicon layer should be thin enough that majority of the strain is confined inside itself and also less bonded with the supporting layer. Several experiments have been conducted in this area, including free-standing nano-membranes[27], fully etched SOI that can be transferred to a host substrate [28, 29], and the backside etching method [30]. However, due to the handling difficulties of nanofilms, it is still highly challenging to obtain an effective compliant substrate that is free of the support constraint and also considerably thinner than the epitaxy layers. Besides, the transfer process tends to introduce contaminations that can significantly degrade the growth quality. As a result, the method for fabricating a suspended silicon substrate from SOI with the thickness under 20 nm is still missing in the literature [30–35].

In this work, an ultrathin ($\sim$16 nm) suspended Si membrane substrate is fabricated by a BOX etching method through hole openings on the top Si layer. This substrate is shown to greatly reduce the mismatches between III-nitride epi-layers and the substrate, and thus greatly improve the crystalline quality of the epi-layers.

## Methods

To understand the influence of top Si layer thickness on dislocation reduction in the epitaxy, the strain distribution between substrate and foreign material epi-layer thickness was investigated. Because of the large lattice and thermal mismatch, there is a significant interfacial strain between the epi-layer and the substrate, resulting in high-density of point defects at the interface [36, 37]. Besides, the high TDDs usually reside in the epi-layers, as the epi-layer is commonly much thinner than the substrate and majority of the strain resides in the epi-layer. In our situation, the substrate is much thinner than the epi-layer, both the substrate and epi-layers can co-accommodate the strain, thus reducing the TDD in epitaxy. According to theory [26, 38–40], the strain of the whole system ($\varepsilon _\mathrm{m}$) and substrate ($\varepsilon _\mathrm{s}$) can be expressed by:

1 $$\begin{aligned} \begin{aligned} \qquad \varepsilon _\text{m}= \varepsilon _\text{s}+\varepsilon _\text{f} \end{aligned} \end{aligned}$$

2 $$\begin{aligned} \begin{aligned} \qquad \varepsilon _\text{s}= \frac{\varepsilon _\text{m} t_\text{f} M_\text{f}}{t_\text{s} M_\text{s}+t_\text{f} M_\text{f}} \end{aligned} \end{aligned}$$

When $\varepsilon _\text{f}$, *M* and *t* represent the epi-layer strain, the biaxial modulus of elasticity and layer thickness, respectively. For an ideal compliant substrate, $t_\text{f} M_\text{f} \gg t_\text{s} M_\text{s}$, $\varepsilon _\text{f} \rightarrow 0$, which requires the substrate thickness $t_\text{s}$ to be smaller than a critical value, so that most of the strain goes to the ultrathin substrate film and the epi-layer can be dislocation-free. According to the critical thickness model [25, 26, 32, 41], the critical thickness of Si film substrate for GaN epitaxy is estimated to be less than 20 nm.

Based on the analysis above, the thickness of Si layer should be reduced to improve the flexibility of a compliant substrate. However, simply thinning the top Si film in SOI structure is insufficient to create an acceptable complaint substrate because the underlying supporting layers are still constraining the Si film [42]. For an effective complaint substrate based on SOI structure, alongside the thin top Si material, the heterojunction interface, between the top Si film and the underneath BOX layer should be very loose [43]. However, the interfacial bond between the Si film and the BOX layer is commonly quite strong, which severely limits the capability of the normal plain SOI in releasing the stress during epitaxy growth. By removing the BOX layer, a suspended structure with better flexibility can be formed, which can release the constraints from all directions.

The suspended substrate as designed was fabricated using the conventional commercial SOI wafer. These SOI substrates were prepared by wafer bonding, consisting a 110 nm silicon (111) top layer, a 670 nm buried oxide layer, and a 740 $\upmu \text{m}$ bottom silicon (100) substrate, as is illustrated in Fig. 1a. To fabricate the suspended substrate, Fig. 1a–d schematically depicts the fabrication flow of the suspended ultrathin silicon membrane substrate. First, the thickness of top silicon was reduced by thermal oxidation thinning process to $\sim$16 nm (Fig. 1b). Second, a series of holes were opened on the top Si film by standard photolithography and inductively coupled plasma (ICP) etching to expose buried oxide layer (Fig. 1c). Those holes are arranged in the shape of a square so that they can prevent stress accumulation in the inner square region of the effectively suspended zone shown in Fig. 1e. Finally, the BOX underneath the top Si film was removed by isotropic etching through those holes using BOE solution (HF: NH$_4$F = 1:6) (Fig. 1d).

The suspended substrates, together with several plain SOI reference substrates, were loaded into a MOCVD reactor (Thomas Swan CCS-MOCVD System) for (Al)GaN growth using trimethylgallium (TMGa), trimethylaluminum (TMAl) and NH$_3$ as precursors. The growth procedure of GaN was divided into three stages: first, 300 nm AlN buffer layer was deposited at a temperature of 1050 $^{\circ }$C and pressure of 60 mbar with the TMAl and NH$_3$ flow rate of 150 and 7500 sccm, respectively. Then, 1 $\upmu \text{m}$ AlGaN was formed at a temperature of 1030 $^{\circ }$C and pressure of 100 mbar with the TMAl, TMGa, and NH$_3$ flow rate of 80, 60 and 15,000 sccm, respectively. Finally, 1.2 $\upmu \text{m}$ GaN layer was grown at a temperature of 1020 $^{\circ }$C and pressure of 300 mbar with the TMGa and NH$_3$ flow rate of 300 and 30,000 sccm, respectively.

## Results and Discussion

**Fig. 2:**
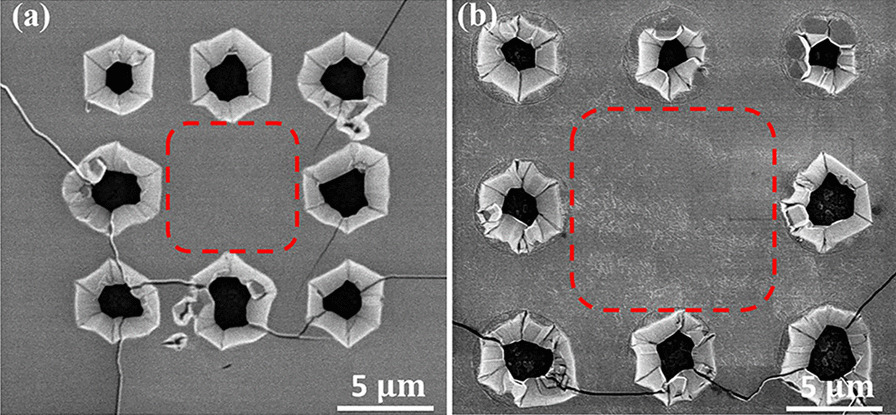
SEM images of (Al)GaN epi-layers on the suspended thin-film substrate with the adjacent hole spacing being **a** 6 $\upmu \text{m}$, **b** 8 $\upmu \text{m}$. The effectively suspended zones are indicated by the red dashed-line squares, which show intact and continuous film quality

The suspended thin-film structures after MOCVD growth were characterized by the scanning electron microscope (SEM). As is shown in Fig. 2, the film cracked preferably along the holes at the suspended region, while the central area of the suspended zones, enclosed by the dashed-lines in Fig. 2, remains intact and displays a continuous film free from cracks. This phenomenon is consistent with our previous research [44], which reveals that during the cooling process, the thermal stress in GaN is heavily accumulated around the holes while well reduced in the effectively suspended zones. As a result, cracks occur only between the holes and the film surface is quite smooth in the effective suspended area. The density of cracks decreased as the adjacent hole spacing increased from 6 to 8 $\upmu \text{m}$ , as shown in Fig. 2a, b, which is in consistent with our simulation results [42]. The holes in this experiment are large, with a diameter in the range of 3–5 $\upmu \text{m}$ , due to the limited capability of experimental tools and the lithograph process. The occurrence of cracking can be further reduced by optimized the hole spacing, hole size and/or hole arrangement.

**Fig. 3:**
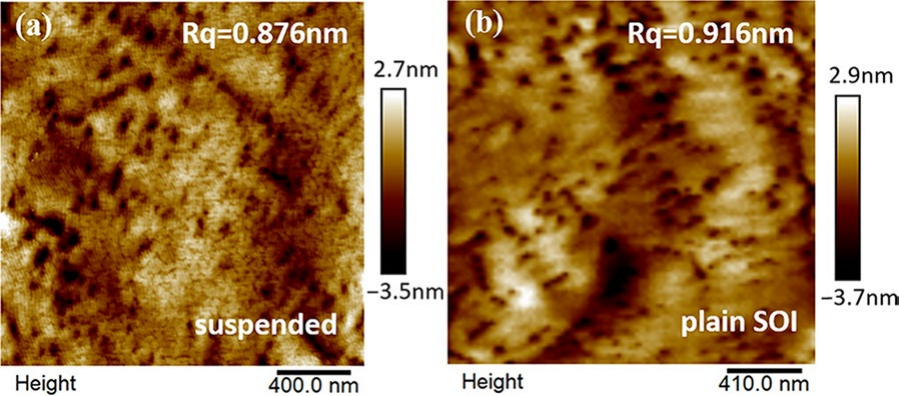
AFM image of GaN surface on **a** effectively suspended zone in the suspended sample, **b** plain SOI sample

Atomic force microscope (AFM) measurement was performed on different locations across the GaN surface of the plain SOI sample, and of the suspended film sample within the effectively suspended zone (Fig. 3). The roughness (Rq) of the GaN epitaxial layer on the plain SOI sample and on the suspended sample, is 0.92 nm and 0.88 nm, respectively. These results indicate that the GaN surface of the effectively suspended zone is smoother than the plain SOI sample, although this surface morphology improvement is quite small.

**Fig. 4:**
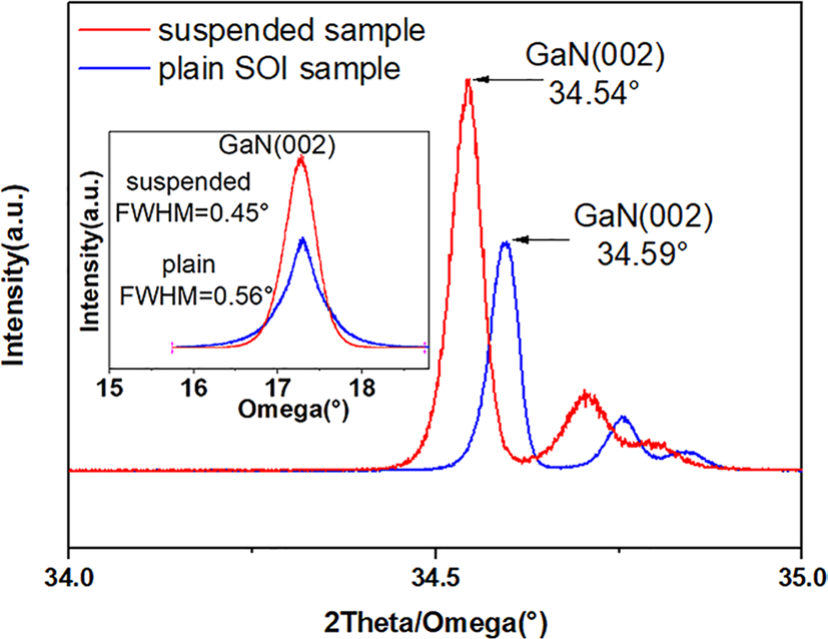
XRD 2theta/omega scan and rocking curves of epitaxial films on suspended sample (red line) and on plain SOI sample (blue line)

To investigate the material quality of GaN epi-layers, X-ray diffraction (XRD) 2theta-omega measurements and rocking curve measurements were taken on both suspended and plain SOI samples with the same growth conditions and thickness. The GaN peak is $\sim$ 34.5$^{\circ }$, which indicates a c-axis orientation, as shown in Fig. 4. The rocking curve FWHM data of GaN on suspended sample (0.45$^{\circ }$) is smaller than on plain SOI (0.56$^{\circ }$), implying that the material quality of epitaxial films grown on the suspended samples is significantly improved. It needs to be noted that X-ray beam spot size (81.25 $\upmu$m $\times$ 12 mm) in the XRD measurement is larger than the effectively suspended zone, and the effective suspended zones account for approximately 10$\%$ of the total measured area. Therefore, the XRD signal includes that from the effectively suspended zone, the surrounding hole areas and non-suspended area. As a result, it can be inferred that GaN epitaxial materials on the effectively suspended zones should have a better crystalline quality than those revealed by XRD characterization.

**Fig. 5:**
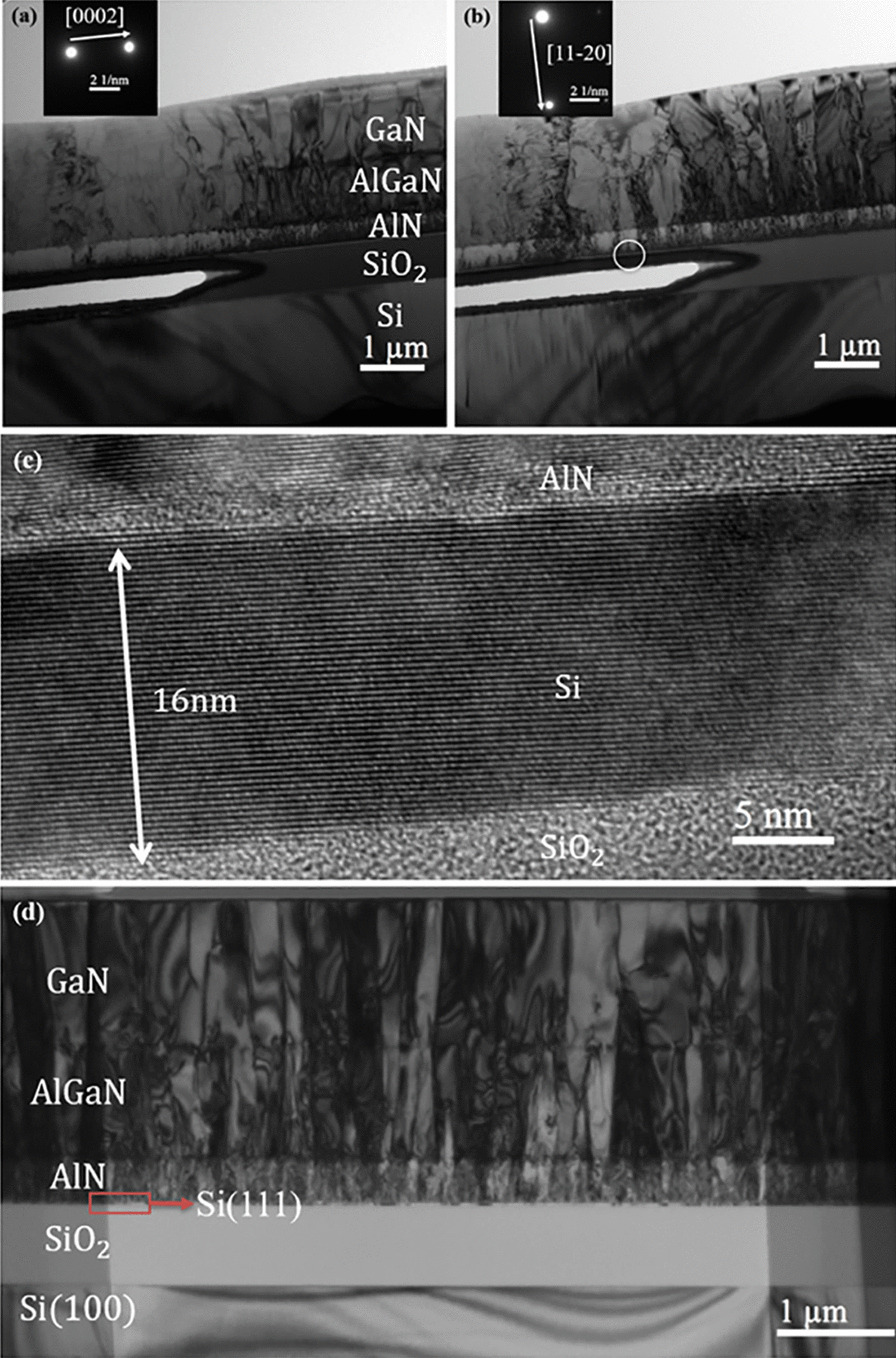
Bright-field cross-sectional TEM images of a suspended ultrathin sample viewed along the [1–100] zone axis with the reflections **a** *g* = [0002] and **b** *g* = [11–20]; the left half of the figures are the fully suspended zones, and right half is non-suspended area. **c** High-resolution TEM image around the circle zone in **b**, indicating the 16 nm thin Si layer between AlN and SiO$_2$ layer. **d** TEM image at the plain SOI regions further away from the right side of the sample

Crystalline quality of the suspended Si nanofilm sample was further investigated by the cross-sectional transmission electron microscope (TEM). Figure 5 shows the bright-field cross-sectional TEM images of a suspended ultrathin sample viewed along the [1–100] zone axis with the reflections (a) *g* = [0002] and (b) *g* = [11–20]. The non-suspension region, shown in right-half region of Fig. 5a, b, contains high-density dislocations. Despite being on the same sample, the dislocation density of the effectively suspended zone at the left half region in Fig. 5a, b appears to be substantially lower than the right half region. TEM image at the plain SOI regions further away from the right side of the sample in Fig. 5a, b were also conducted, which also exhibits significantly higher threading dislocation density than that in the suspended region, as shown in Fig. 5d. That the crystalline quality of the effectively suspended zone (left half) is significantly better than that of the non-suspended regions is a strong evidence for the effectiveness of suspended ultrathin Si nanofilm structure in strain relief.

According to the prior analysis, when the suspended Si substrate is substantially thinner than the epi-layer, almost all of the strain is confined in the ultrathin Si layer. Despite this, no dislocation generation is observed in the ultrathin Si layer when it was inspected by high-resolution TEM (HRTEM), which can be explained by the remarkable flexibility of a nanofilm. The top Si film in our suspended ultrathin samples is only 16 nm thick as shown in Fig. 5c, which can tolerate most strain and prevent the formation of defects.

**Fig. 6:**
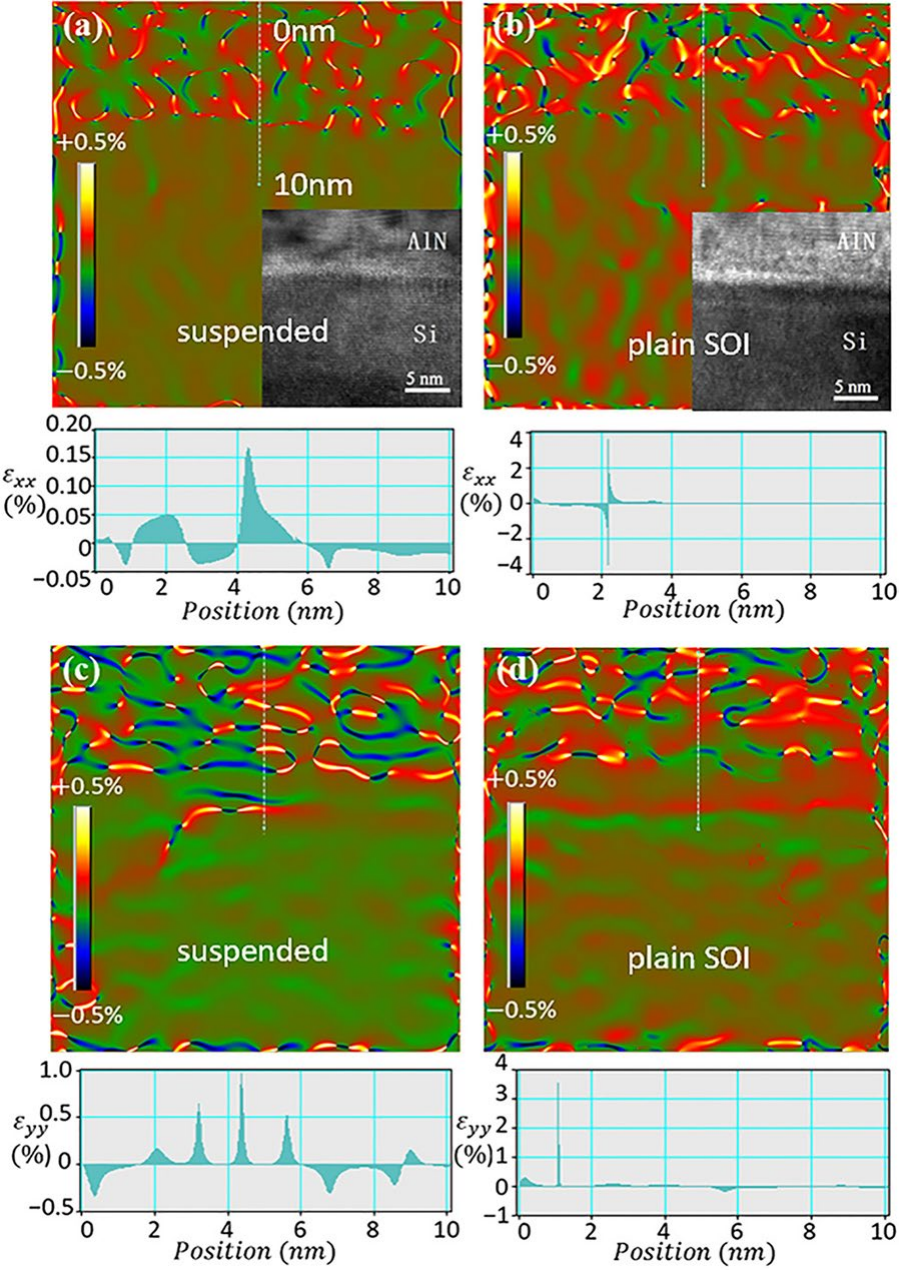
HRTEM images of the effectively suspended zone, and the strain map distribution (**a**, **c**). HRTEM images of plain SOI sample, and the strain map distribution (**b**, **d**). The strain map distribution and its respective vertical line profile along the $\varepsilon _{xx}$ direction (**a**, **b**), and the $\varepsilon _{yy}$ direction (**c**, **d**). In each image, the GPA maps are generated by defining an unstrained reference area in the Si layer, and the strain values are the relative value of the compressed Si lattice. To emphasize the contrast at the Si area, the color scale was tuned at $-0.5\%$ to +0.5$\%$

HRTEM images of GaN epitaxy on the effectively suspended zone and the plain SOI samples were analyzed. As the lattice parameter of unstrained AlN ($a=3.110 \,\text{\AA}$, $c=4.978\, \text{\AA}$ ) is smaller than that of unstrained Si (5.428 $\text{\AA}$ ), Si nanofilm substrate is subjected to compressive stress. Based on the measurement and calculation on the diffraction patterns, the estimated lattice constant of the top Si layer for the suspended nanofilm sample is 5.265 $\text{\AA}$ and that for the plain SOI sample is 5.374 $\text{\AA}$ . This shows that the suspended Si nanofilm is more flexible and hence compressed more (Table 1). As a result, dislocations can be significantly reduced during epitaxy growth by the strain release in suspended ultrathin Si substrate, which is in accordance with the observation shown in the cross-sectional TEM images and previous studies. Figure 6 shows the geometric phase analysis (GPA) of strain values conducted using the HRTEM image around the interface between AlN and Si in the horizontal ($\varepsilon _{xx}$) and vertical ($\varepsilon _{yy}$) directions, and a vertically measured strain line profile at the dashed line of the image. As shown in Fig. 6a, c, the strain distribution on the *x* and *y* direction in the effectively suspended zone has a uniform strain distribution, with relative strain values ranging from $-0.5$ to +1$\%$. In contrast, the plain SOI sample with the same Si thickness has a much larger strain variation with a value up to +4.0$\%$ as shown in Fig. 6b, d. The more uniform strain distribution in the suspended structure is also implying enhanced strain release.

**Table 1:** Lattice parameter of Si layer near the AlN/Si interface

Material	Unstrained Si	Suspended Si nanofilm	Top Si layer on SOI
Lattice constant (Å)	5.428	5.265	5.374

## Conclusions

In summary, an ultrathin suspended Si nanofilm substrate has been developed for the epitaxial growth of high mismatch materials. The suspended structure complaint substrate with an ultrathin nanofilm allows for the effective release of stress induced by the lattice and thermal mismatches. (Al)GaN layers grown on the effectively suspended zone has a continuous integrity, better surface smoothness, and greatly reduced dislocations. Because the stress of GaN epi-layer on the effectively suspended zone is well released, the crystal quality of GaN epi-layers on the suspended ultrathin Si nanofilm is improved significantly. This suspended substrate fabrication strategy is cost-effective and easy to fabricate compared with other strategies in obtaining an effective compliant substrate. Thus, it offers a cost-effective way to integrate nitride device into silicon-based integrated circuits for more advanced functions. Furthermore, this study can also be very helpful in the growth of a wide variety of materials that needs to deal with a large strain.

## Data Availability

The data set used and/or analyzed in this study can be obtained from the corresponding author upon reasonable request.

## Abbreviations

GaN: Gallium nitride;
SOI: Silicon on insulator;
AlN: Aluminum nitride;
TEM: Transmission electron microscope;
HRTEM: High-resolution transmission electron microscope;
TDD: Threading dislocation density;
BOX: Buried oxide layer;
SIMOX: Separation by implantation of oxygen;
ICP: Inductively coupled plasma;
BOE: Buffered oxide etch;
MOCVD: Metal-organic chemical vapor deposition;
CCS-MOCVD: Closed coupled showerhead metal-organic chemical vapor deposition;
TMGa: Trimethylgallium;
TMAl: Trimethylaluminum;
SEM: Scanning electron microscope;
AFM: Stomic force microscope;
XRD: X-ray diffraction.
